# Lipid Metabolic Reprogramming and Epigenetic Co-Dysregulation Across the Central Chondrosarcoma Grade Spectrum: A Multi-Cohort RNA-seq Study

**DOI:** 10.3390/ijms27125307

**Published:** 2026-06-11

**Authors:** Batuhan Ayhan, Neslihan Dönmez, Zeliha Deniz Ayhan

**Affiliations:** 1 Department of Orthopaedics and Traumatology, Haymana State Hospital, Haymana, 06860 Ankara, Türkiye; 2 Department of Bioinformatics, Graduate School of Health Sciences, Hacettepe University, 06100 Ankara, Türkiye; 3 Department of Medical Pathology, Faculty of Medicine, Gazi University, 06560 Ankara, Türkiye

**Keywords:** chondrosarcoma, lipid metabolic reprogramming, epigenetic dysregulation, bioinformatics, *SQLE*, *FASN*, *ACACA*, single-cell RNA-seq

## Abstract

Central chondrosarcoma is the second most common primary malignant bone tumour, and grade progression markedly worsens prognosis. The contributions of lipid metabolic reprogramming and epigenetic co-dysregulation to grade progression remain poorly characterised. We integrated a bulk RNA-seq discovery cohort of 53 graded central chondrosarcomas (GSE299759) with a single-cell analysis of eight chondrosarcomas (GSE184118). Because the atypical cartilaginous tumour (ACT) and dedifferentiated groups each comprised only three samples, the Grade 3 versus Grade 2 contrast was pre-specified as the primary comparison. Curated panels of 44 lipid metabolism genes and 50 epigenetic regulators were assessed by differential expression and a correlation-based connectivity ranking, evaluated by permutation testing. In the primary Grade 3 versus Grade 2 comparison, *SQLE*, *ACACA*, and *FASN* were upregulated (FDR < 0.05), indicating a grade-associated increase in de novo lipogenesis. In the exploratory Grade 3 versus ACT comparison, additional lipid genes (*HMGCR*, *LDLRAP1*) and the epigenetic regulators *EHMT2* and *SIRT2* showed altered expression, although the small ACT group limits these estimates. A connectivity ranking highlighted *FASN*, *KMT2C*, *TET2*, *SETD5*, and *KDM5B*; permutation testing confirmed this co-expression structure was non-random (*p* < 0.0001). Single-cell analysis showed *FASN*, *SETD5*, and *KDM5B* are expressed predominantly in malignant cells, whereas *KMT2C* and *TET2* are not, indicating cell-type heterogeneity. De novo lipogenesis upregulation is the most consistent lipid alteration in high-grade central chondrosarcoma, nominating *SQLE*, *ACACA*, and *FASN* as candidates for experimental investigation.

## 1. Introduction

Chondrosarcoma is the second most common primary malignant bone tumour, representing 20–27% of all malignant primary bone neoplasms [1]. The vast majority (85%) are conventional central chondrosarcomas (CCSs), which arise within the medullary cavity and are graded on a three-tier scale: Grade 1 (reclassified as atypical cartilaginous tumour, ACT, in appendicular locations per the 2020 WHO Classification [2]), Grade 2, and Grade 3. Dedifferentiated chondrosarcoma (DDCS) represents a distinct high-grade variant carrying a 5-year overall survival of only 7–24% [3,4].

Histological grading remains the strongest predictor of metastatic potential and survival. *IDH1*/2 mutations occur in 40–70% of conventional CCSs [5], and *IDH* status has independent prognostic value [6], generating the oncometabolite D-2-hydroxyglutarate (D-2HG), which inhibits TET-family DNA demethylases and JmjC-domain histone demethylases, resulting in a global CpG island methylator phenotype and altered histone landscapes [7,8]. This mechanistic link positions epigenetic dysregulation as a fundamental driver of chondrosarcoma genesis.

Lipid metabolic reprogramming is an established hallmark of cancer [9], increasingly recognised as a co-driver of malignant progression in sarcomas and other mesenchymal tumours [10]. *FASN*, *ACACA*, *HMGCR*, *SQLE*, and *PPARG* are dysregulated in high-grade mesenchymal tumours [11,12,13,14], and *SIRT1*—linking metabolic sensing to epigenetic control—is overexpressed in high-grade and dedifferentiated chondrosarcoma [15]. D-2HG further rewires lipid biosynthesis via altered acetyl-CoA availability, suggesting an *IDH*–lipid–epigenetic axis unexplored in this tumour type.

The recent deposition of a bulk RNA-seq dataset (GSE299759; [7]) from 54 graded central chondrosarcoma patients provides an opportunity for systematic characterisation, building on prior multi-omics molecular profiling of this tumour type [16]. Complementary single-cell RNA-seq data from conventional central chondrosarcomas (GSE184118; [17]) additionally allow a cell-type-resolved view of these transcriptomic findings. In this study, we (i) characterise the global transcriptome architecture across grades, (ii) assess the grade-stratified expression of curated lipid and epigenetic gene panels, (iii) compute composite expression scores, (iv) rank hub genes at the lipid–epigenetic interface using a co-expression analysis, and (v) resolve the cell-type distribution of these hub genes by single-cell RNA-seq across eight chondrosarcomas.

## 2. Results

### 2.1. Cohort Characteristics

The composition of the bulk RNA-seq discovery cohort, including grade distribution and key sequencing metrics, is summarised in Table 1; full data-processing details for both the bulk RNA-seq discovery cohort and the single-cell cohort are provided in the Materials and Methods (Section 4.2 and Section 4.3). As noted there, the bulk discovery cohort is markedly unbalanced, with the ACT and DDCS groups comprising only three samples each.

### 2.2. Global Transcriptome Architecture Across Grade

**Figure 1:**
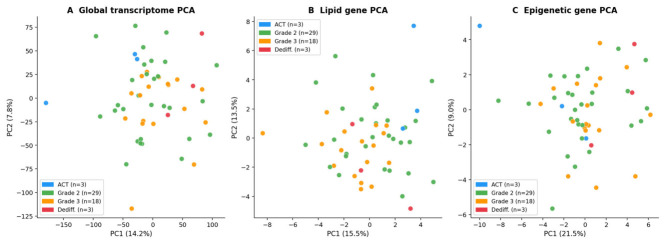
Principal component analysis of GSE299759 (*n* = 53). (**A**) Global transcriptome PCA (19,616 genes). (**B**) Lipid gene PCA (44 genes). (**C**) Epigenetic gene PCA (50 genes). Colour: blue = ACT (*n* = 3), green = Grade 2 (*n* = 29), orange = Grade 3 (*n* = 18), red = DDCS (*n* = 3). The percentage variance explained is shown in parentheses.

The PCA of all 19,616 expressed genes (Figure 1A) revealed that PC1 (14.2% variance) separated DDCS samples from conventional grades, while PC2 (7.8%) further distinguished ACT from Grades 2 and 3. The grade-restricted PCA of the 44-gene lipid panel (Figure 1B) and 50-gene epigenetic panel (Figure 1C) each confirmed this topology; thus, both modules carry independent grade-informative signals.

### 2.3. Grade-Stratified Lipid Gene Expression

**Figure 2:**
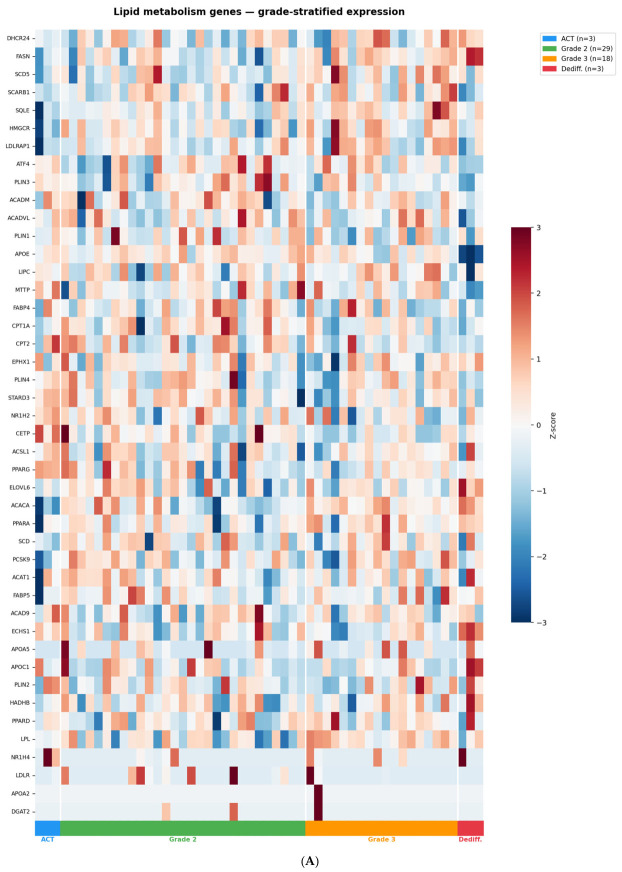

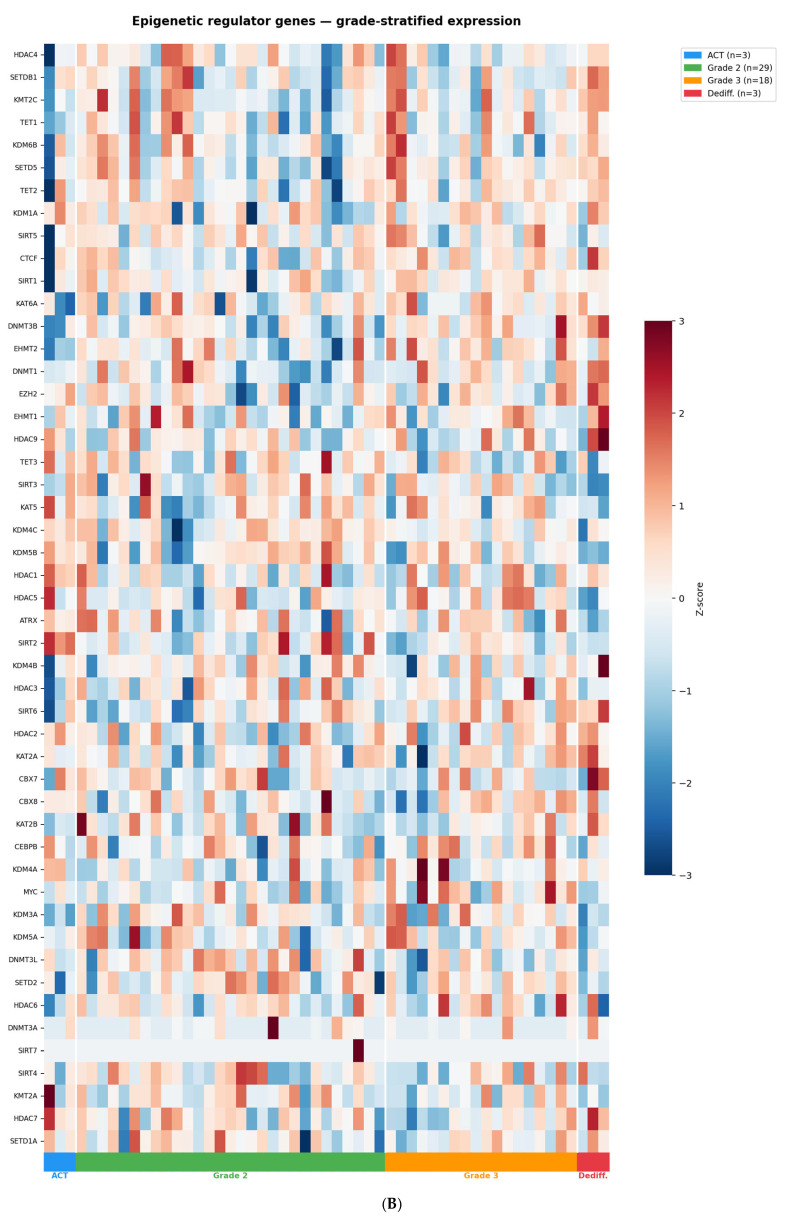
(**A**) Lipid metabolism gene expression heatmap (44 genes, *n* = 53 samples). Rows: genes ordered by Ward-linkage clustering. Columns: samples ordered by grade (ACT → Grade 2 → Grade 3 → DDCS) with within-grade clustering. Colour scale: z-scored log_2_-CPM, clipped to ±3 (red = high, blue = low). Grade colour bar above: blue = ACT, green = Grade 2, orange = Grade 3, red = DDCS. (**B**) Epigenetic regulator gene expression heatmap (50 genes, *n* = 53 samples). Layout and colour scale as in (**A**).

Hierarchical clustering of the lipid expression matrix (Figure 2A) revealed a progressive upregulation of fatty acid synthesis and cholesterol biosynthesis genes (*FASN*, *ACACA*, *SQLE*, *SCD5*) from ACT to Grade 3 and downregulation of lipid-sensing receptors (*PPARG*, *PPARA*) and apolipoproteins (*APOE*) in higher-grade tumours.

In the primary, adequately powered Grade 3 vs. Grade 2 comparison (*n* = 18 vs. *n* = 29), Dunn post hoc analysis (following Kruskal–Wallis omnibus testing across all four grades) identified three significant lipid genes at FDR < 0.05: *SQLE* (squalene epoxidase; log_2_FC = +0.82, *p*adj = 0.012), *ACACA* (acetyl-CoA carboxylase; *p*adj = 0.017), and *FASN* (*p*adj = 0.032), with *SCD5* reaching the relaxed exploratory threshold of FDR < 0.10 (*p*adj = 0.058). These findings constitute the confirmatory result of the study and indicate a grade-associated upregulation of de novo lipogenesis and cholesterol biosynthesis in this contrast. The remaining pairwise comparisons involve the ACT or DDCS groups (*n* = 3 each) and are reported as exploratory and underpowered. In the exploratory Grade 3 vs. ACT comparison, the following genes were nominally significant at FDR < 0.05: *SQLE* (log_2_FC = +3.02, *p*adj = 0.008), *ACACA* (log_2_FC = +1.18, *p*adj = 0.004), *HMGCR* (HMG-CoA reductase; log_2_FC = +1.49, *p*adj = 0.033), and *LDLRAP1* (log_2_FC = +3.05, *p*adj = 0.049); at the relaxed FDR < 0.10 threshold, *CETP* (log_2_FC = −1.29, *p*adj = 0.094), *PPARG* (log_2_FC = −1.34, *p*adj = 0.083), *SCD5* (log_2_FC = +1.19, *p*adj = 0.100), and *FASN* (log_2_FC = +0.95, *p*adj = 0.059) were additionally flagged. Because the ACT group comprises only three samples, the large fold-change estimates in this contrast (e.g., *SQLE* +3.02, *LDLRAP1* +3.05) are unstable and should be regarded as hypothesis-generating rather than reliable effect sizes. The directional consistency of *SQLE*, *ACACA*, and *FASN* between the exploratory Grade 3 vs. ACT contrast and the primary Grade 3 vs. Grade 2 contrast is noted as supportive but not independent confirmatory evidence, since the two contrasts share the Grade 3 samples.

### 2.4. Grade-Stratified Epigenetic Regulator Expression

Hierarchical clustering of the epigenetic regulator matrix (Figure 2B) revealed complex grade-associated patterns. Dunn post hoc analysis identified *EHMT2* (H3K9 methyltransferase; log_2_FC = +1.00, *p*adj = 0.014) and *SIRT2* (NAD^+^-dependent deacetylase; log_2_FC = −1.06, *p*adj = 0.035) as significantly altered at FDR < 0.05 in Grade 3 vs. ACT. *KDM3A* (log_2_FC = +0.71, *p*adj = 0.059) and *DNMT3B* (log_2_FC = +1.35, *p*adj = 0.092) reached FDR < 0.10. In the Grade 3 vs. Grade 2 comparison, no epigenetic gene survived FDR < 0.05, but *EHMT2* (*p*adj = 0.081) and *DNMT3B* (*p*adj = 0.098) showed directionally consistent trends at FDR < 0.10. Active chromatin regulators (*KMT2C*, *TET2*, *SETD5*) did not reach significance in pairwise comparisons but emerged as the top hub genes by co-expression connectivity (Section 2.7).

### 2.5. Composite Expression Scores by Grade

**Figure 3:**
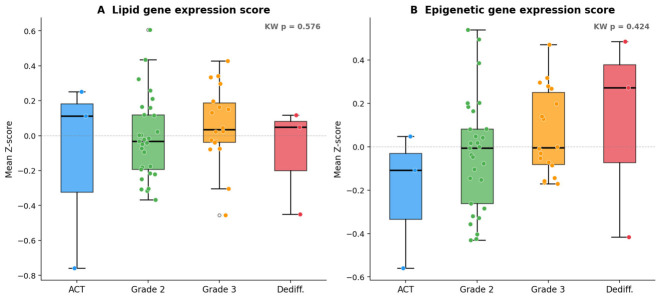
Composite expression score boxplots by histological grade. (**A**) Lipid gene expression score. (**B**) Epigenetic regulator expression score. Scores represent mean z-scored log_2_-CPM across all panel genes per sample. Individual samples overlaid as jittered points. Kruskal–Wallis *p*-values shown. Colour as in Figure 1.

The composite expression scores showed non-significant trends across grade groups for both the lipid panel (Kruskal–Wallis *p* = 0.576; Figure 3A) and the epigenetic panel (*p* = 0.424; Figure 3B). The absence of significant group-level differences reflects the within-grade heterogeneity and the small ACT and DDCS sample sizes, rather than an absence of directional gene-level signals.

### 2.6. Ranked Expression Profiles of Pairwise Differential Expression

**Figure 4:**
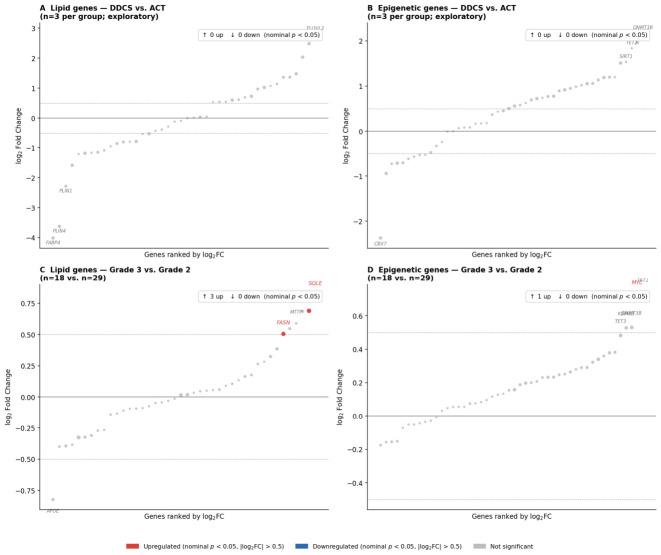
Ranked dot plots of pairwise differential expression analyses. Genes are ordered along the *x*-axis by log_2_ fold change; the dot size reflects the −log_10_(nominal *p*-value). Coloured dots indicate nominal *p* < 0.05 and |log_2_FC| > 0.5 (red: upregulated; blue: downregulated); grey: not significant. Dashed horizontal lines mark |log_2_FC| = 0.5. (**A**) Lipid genes—DDCS vs. ACT (*n* = 3 per group; exploratory). (**B**) Epigenetic genes—DDCS vs. ACT (exploratory). (**C**) Lipid genes—Grade 3 vs. Grade 2 (*n* = 18 vs. *n* = 29). (**D**) Epigenetic genes—Grade 3 vs. Grade 2. Top genes by magnitude labelled on each panel.

Ranked dot plots (Figure 4) display all tested genes ordered by log_2_ fold change, with the dot size proportional to −log_10_(nominal *p*-value). Coloured dots indicate nominal *p* < 0.05 and |log_2_FC| > 0.5; BH-corrected significance values are reported in Table 2. In the DDCS vs. ACT comparisons (Figure 4A,B), no genes reached statistical significance after correction, in the context of *n* = 3 per group. Directionally, *APOE* showed the largest magnitude lipid gene difference (log_2_FC = −5.88 in DDCS vs. ACT). In the Grade 3 vs. Grade 2 lipid comparison (Figure 4C), cholesterol biosynthesis and lipogenic genes (*SQLE*, *FASN*, *ACACA*, *SCD5*) clustered at the positive extreme. The Grade 3 vs. Grade 2 epigenetic comparison (Figure 4D) showed *KDM4A* and *MYC* at the positive extreme and *DNMT3L* at the negative extreme.

### 2.7. Hub Gene Identification: Lipid–Epigenetic Interface

**Figure 5:**
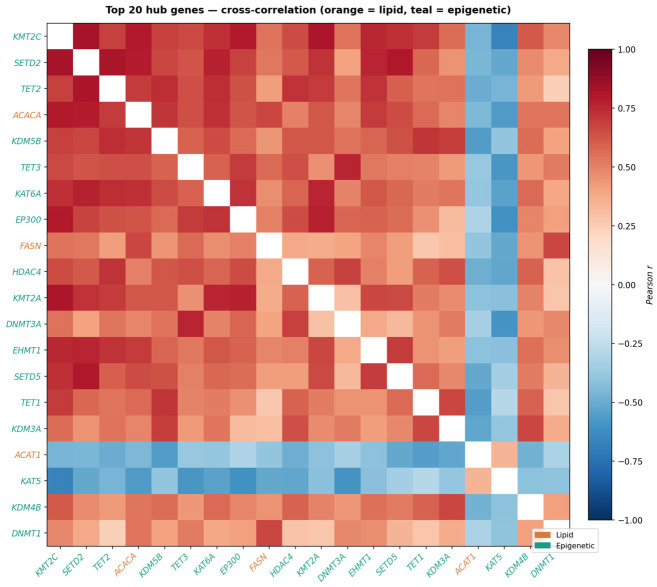
Cross-correlation matrix of the top 20 hub genes from the combined 94-gene lipid–epigenetic co-expression matrix. Colour represents Pearson r (red = positive, blue = negative). Gene names are coloured by module: orange = lipid metabolism, teal = epigenetic regulator. Self-correlations (diagonal) are masked. Genes are ordered by hub score (mean |r| with all other matrix genes).

**Figure 6:**
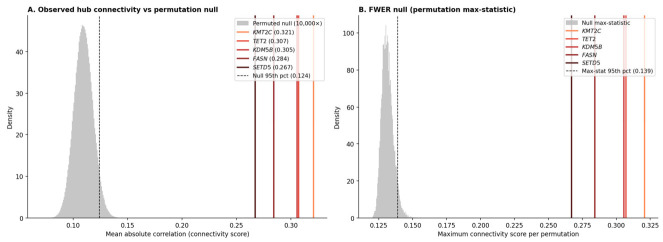
Permutation test of hub gene connectivity. Gene labels in the 94-gene lipid–epigenetic matrix were independently permuted across samples 10,000 times to generate a null distribution of connectivity scores under the assumption of no inter-gene correlation structure. (**A**) Pooled null distribution of connectivity scores (grey) with the observed connectivity of the five hub genes (vertical lines); the dashed line marks the 95th percentile of the null. (**B**) Null distribution of the per-permutation maximum connectivity score (family-wise error control); all five hub genes exceed the 95th percentile of this stringent null. All five hub genes had empirical and family-wise error-corrected *p* < 0.0001, indicating that their high connectivity is not attributable to chance.

To prioritise genes at the lipid–epigenetic interface for further consideration, an exploratory connectivity ranking was computed: each gene in the combined 94-gene lipid–epigenetic matrix was assigned a connectivity score equal to its mean absolute Pearson correlation coefficient (|r|) with all other 93 genes in the matrix, encompassing both within-module (lipid–lipid and epigenetic–epigenetic) and between-module (lipid–epigenetic) correlations, and genes were ranked by this score (Table 3; Figure 5). The five highest-ranked genes were *KMT2C* (connectivity score 0.282), *KDM5B* (0.271), *SETD5* (0.267), *TET2* (0.262), and *FASN* (0.257). It should be emphasised that these connectivity scores are modest in absolute terms (mean |r| ≈ 0.26–0.28), so the ranking distinguishes relatively more connected genes from less connected ones rather than identifying strong correlations; no fixed |r| threshold was applied to define significant interactions. Although the absolute correlations are modest, a permutation test (10,000 permutations) showed that the connectivity scores of the top-ranked genes are nonetheless far higher than expected by chance: the permutation null distribution had a mean connectivity of 0.111 and a 95th percentile of 0.125, whereas all five top-ranked genes (*KMT2C*, *KDM5B*, *SETD5*, *TET2*, *FASN*) had observed scores of 0.26–0.32, corresponding to empirical and family-wise error-corrected *p*-values < 0.0001 (Figure 6). The permutation test therefore indicates that the co-expression structure underlying the ranking is statistically robust and not an artefact of random correlation, even though the magnitude of individual correlations is modest. Accordingly, this analysis is descriptive and hypothesis-generating, and the co-ranking of the lipid gene *FASN* alongside the epigenetic regulators *KMT2C* and *TET2* should be regarded as a statistically supported transcriptional association to be tested experimentally, not as evidence of a direct molecular interaction.

### 2.8. Single-Cell Analysis Across Eight Chondrosarcomas: Cell-Type Distribution of Hub Genes (GSE184118)

**Figure 7:**
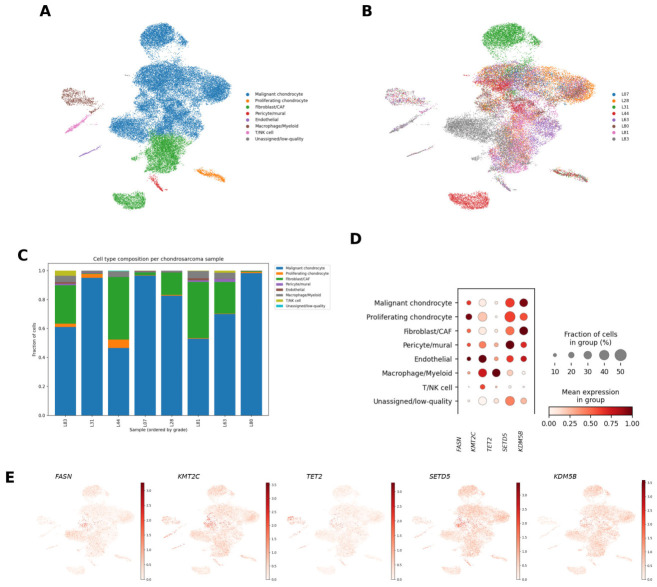
Single-cell RNA-seq analysis of hub gene expression across eight chondrosarcomas (GSE184118; 36,012 quality-controlled cells). (**A**) UMAP embedding of all cells after Harmony integration, coloured by annotated cell type. (**B**) The same UMAP coloured by sample of origin, showing that the eight samples are well mixed after integration. (**C**) Stacked bar chart of cell-type composition per sample, ordered by histological grade, illustrating substantial inter-tumour variation in microenvironmental composition. (**D**) Dot plot of the five bulk-derived hub genes (*FASN*, *KMT2C*, *TET2*, *SETD5*, *KDM5B*) across cell types; dot size indicates the fraction of expressing cells and colour indicates scaled mean expression. (**E**) UMAP feature plots of the five hub genes (log-normalised expression). *FASN*, *SETD5*, and *KDM5B* are expressed predominantly in malignant chondrocytes, whereas *TET2* is expressed mainly in macrophage/myeloid cells and *KMT2C* is not enriched in tumour cells.

To resolve the cell-type distribution of the bulk-derived hub genes, we analysed single-cell RNA-seq data from all eight chondrosarcoma samples in GSE184118 ([17]). After uniform quality control, 36,012 cells were retained (range 2243–6810 per sample). Following log-normalisation, batch-aware highly variable gene selection, principal component analysis, and Harmony integration across the eight samples, Leiden clustering with canonical marker-gene annotation identified the expected populations of the chondrosarcoma tumour microenvironment (Figure 7A,B): malignant chondrocytes (26,358 cells, 73.2%), a smaller proliferating chondrocyte cluster (618 cells, 1.7%; MKI67/TOP2A-positive), fibroblasts/cancer-associated fibroblasts (CAFs; 7272 cells, 20.2%), macrophage/myeloid cells (972 cells, 2.7%), and minor pericyte/mural, endothelial, and T/NK-cell populations (each < 1%). The malignant chondrocyte fraction varied substantially between patients (46.6–98.2%), confirming that inter-tumour heterogeneity in microenvironmental composition is appreciable and underlining the value of analysing multiple samples rather than one (Figure 7C).

We then examined the five bulk-derived hub genes (*FASN*, *KMT2C*, *SETD5*, *TET2*, *KDM5B*) across cell types. Three of the five showed significantly higher expression in malignant cells (malignant chondrocytes plus proliferating chondrocytes) than in non-malignant cells: *FASN* (Mann–Whitney U, *p* = 2.6 × 10^−12^), *SETD5* (*p* = 8.3 × 10^−21^), and *KDM5B* (*p* = 4.5 × 10^−6^), indicating that these three genes are expressed in a tumour-cell-predominant manner. The remaining two hub genes did not follow this pattern. *KMT2C* was not enriched in malignant cells (*p* = 0.99), being expressed at comparable or slightly higher levels in endothelial and myeloid cells. *TET2* was expressed at its highest level in macrophage/myeloid cells (mean 0.327 log-normalised units) and was comparatively low in malignant chondrocytes (mean 0.046). Thus the single-cell data support tumour-cell-predominant expression for the lipid hub gene *FASN* and for *SETD5* and *KDM5B*, but indicate that *KMT2C* and—particularly—*TET2* are not predominantly tumour-cell-derived (Figure 7D,E).

Restricting attention to the malignant chondrocyte compartment and stratifying by the GEO grade label of each sample, hub gene expression did not show a consistent monotonic increase with grade; for example, mean *FASN* expression in malignant cells was 0.054, 0.074, 0.066, and 0.182 log-normalised units in low-, intermediate-, high-grade, and dedifferentiated samples, respectively. This single-cell grade comparison should be interpreted cautiously, because the eight samples are distributed unevenly across grade categories and droplet-based scRNA-seq is subject to substantial gene dropout (the hub genes were detected in only 5–44% of cells, depending on gene and cell type). Overall, the multi-sample single-cell analysis localises *FASN*, *SETD5*, and *KDM5B* expression predominantly to tumour cells, but does not by itself establish a grade-dependent gradient at single-cell resolution.

## 3. Discussion

This bioinformatic study examined lipid metabolic and epigenetic transcriptional differences across the central chondrosarcoma grade spectrum using Kruskal–Wallis omnibus testing followed by Dunn post hoc pairwise analysis. We distinguish here between confirmatory and exploratory findings. The single confirmatory finding, derived from the adequately powered Grade 3 vs. Grade 2 comparison (*n* = 18 vs. *n* = 29), is a grade-associated upregulation of de novo lipogenesis and cholesterol biosynthesis genes (*SQLE*, *ACACA*, *FASN*) at FDR < 0.05. All remaining observations are exploratory and should be interpreted with caution: (i) the additional lipid genes implicated only in comparisons involving the ACT or DDCS groups (e.g., *HMGCR* and *LDLRAP1* in Grade 3 vs. ACT), where each group comprised only three samples; (ii) the epigenetic differences (*EHMT2*, *SIRT2*) seen at FDR < 0.05 in the underpowered Grade 3 vs. ACT contrast; (iii) the lipid–epigenetic connectivity ranking, which—although shown by permutation testing to be statistically robust—remains correlative and is not a test of mechanism; and (iv) the single-cell analysis of eight chondrosarcomas, which localises hub gene expression by cell type but does not establish a grade-dependent gradient at single-cell resolution. The discussion below is organised accordingly, and claims that depend on the ACT or DDCS groups, on the connectivity ranking, or on the single-cell analysis are framed as hypotheses for future testing rather than as established conclusions.

### 3.1. SQLE, ACACA, and FASN: Grade-Associated De Novo Lipogenesis Upregulation

Considering first the confirmatory finding introduced above, the Grade 3 vs. Grade 2 contrast showed grade-associated upregulation of *SQLE* (squalene epoxidase), *ACACA* (acetyl-CoA carboxylase), and *FASN*, all at FDR < 0.05. The same three genes were also nominally significant in the exploratory Grade 3 vs. ACT comparison (*SQLE* log_2_FC = +3.02, FDR = 0.008; *ACACA* log_2_FC = +1.18, FDR = 0.004); however, because the ACT group comprises only three samples, the large fold-change estimates from that contrast are unstable and are regarded here as hypothesis-generating rather than reliable effect sizes, and the directional agreement between the two contrasts is noted as supportive but not independent confirmation, since they share the Grade 3 samples. Mechanistically, *SQLE* catalyses a rate-limiting step in cholesterol biosynthesis, and its overexpression has been reported as a driver of aggressive behaviour across several cancer types including hepatocellular carcinoma, colorectal cancer, and head and neck squamous cell carcinoma [18,19,20]. *ACACA* converts acetyl-CoA to malonyl-CoA, the committed step of de novo fatty acid synthesis, and acts upstream of *FASN* in the lipogenic cascade [14]. The coordinated upregulation of *ACACA*, *FASN*, and *SQLE* between Grade 2 and Grade 3 is consistent with a broad activation of lipogenic and sterologenic metabolism, which may reflect the increased anabolic demand of proliferating high-grade tumour cells [9]. *SQLE* is pharmacologically targetable with terbinafine and *ACACA* with ND-646 or TOFA; on the basis of these transcriptomic associations, both enzymes are nominated as candidate targets for future pre-clinical evaluation in high-grade chondrosarcoma, although such proposals remain hypotheses requiring experimental testing.

### 3.2. HMGCR and LDLRAP1: Cholesterol Biosynthesis and Uptake

*HMGCR* (HMG-CoA reductase) and *LDLRAP1* (LDL receptor adaptor protein 1) were nominally significant only in the exploratory Grade 3 vs. ACT comparison (*HMGCR* log_2_FC = +1.49, FDR = 0.033; *LDLRAP1* log_2_FC = +3.05, FDR = 0.049) and did not reach significance in the primary Grade 3 vs. Grade 2 contrast. Because the ACT group comprises only three samples, these results are underpowered and the corresponding fold-change estimates are unstable; the observations below are therefore presented as exploratory and hypothesis-generating rather than as established grade-associated changes. *HMGCR* is the rate-limiting enzyme of the mevalonate pathway and the target of statin drugs, and is regulated by SREBP-2 in response to intracellular sterol depletion [21,22]. If confirmed in adequately powered cohorts, an upregulation of *HMGCR* alongside *SQLE* could be consistent with a coordinated activation of the cholesterol biosynthetic axis, from mevalonate production to squalene epoxidation. Statins have shown anti-proliferative activity in osteosarcoma models [19]; the present exploratory data may, with this caveat, provide a preliminary transcriptomic rationale for examining mevalonate pathway inhibition in high-grade central chondrosarcoma. *LDLRAP1* mediates clathrin-dependent internalisation of the LDL receptor and thereby facilitates exogenous cholesterol uptake; its possible upregulation, if validated, would be compatible with the hypothesis that high-grade chondrosarcoma exploits both de novo synthesis and lipoprotein uptake to meet sterol demand. All of these interpretations require confirmation in larger cohorts before any mechanistic or therapeutic conclusion can be drawn.

### 3.3. PPARG Downregulation and Loss of Lipid Catabolism

*PPARG* showed reduced expression in the exploratory Grade 3 vs. ACT comparison, reaching only the relaxed secondary threshold (log_2_FC = −1.34, FDR = 0.083; FDR < 0.10) and not the primary FDR < 0.05 criterion; it was not significant in the primary Grade 3 vs. Grade 2 contrast. This observation is therefore exploratory and underpowered. With that caveat, *PPARG* is a regulator of lipid catabolism, adipogenesis, and anti-inflammatory signalling, and a reduction in its expression in higher-grade tumours could be consistent with a loss of chondrocyte differentiation programmes and of a metabolic brake on anabolic lipogenesis [23]. The apparent directional pattern—reduced *PPARG* together with the grade-associated increase in *ACACA*, *FASN*, and *SQLE*—may be compatible with a shift from catabolic toward anabolic lipid metabolism, but this interpretation is hypothesis-generating and would need to be tested in an adequately powered Grade 2 vs. Grade 3 comparison. *PPARG* agonists have shown anti-tumour activity in sarcoma cell lines [23]; the expression pattern observed here warrants further, appropriately powered investigation before any conclusion is drawn.

### 3.4. EHMT2 and SIRT2: Epigenetic Reprogramming in Grade 3

Two epigenetic regulators, *EHMT2* (G9a; H3K9 dimethyltransferase; log_2_FC = +1.00, FDR = 0.014) and *SIRT2* (NAD^+^-dependent deacetylase; log_2_FC = −1.06, FDR = 0.035), reached FDR < 0.05 in the exploratory Grade 3 vs. ACT comparison. It is important to note that, in the primary and adequately powered Grade 3 vs. Grade 2 comparison, no epigenetic regulator reached FDR < 0.05; only *EHMT2* (FDR = 0.081) and *DNMT3B* (FDR = 0.098) showed directionally consistent trends at the relaxed FDR < 0.10 threshold. The epigenetic differences described here are therefore exploratory and underpowered, and should be read as candidate observations rather than confirmed grade-associated changes. With this caveat, *EHMT2* catalyses H3K9me1/2, a modification associated with transcriptional repression and heterochromatin formation; if its apparent upregulation were confirmed, it could be compatible with enhanced silencing of tumour suppressor or differentiation loci, in line with epigenetic silencing programmes reported in high-grade bone sarcomas [24,25]. *SIRT2* is a cytoplasmic NAD^+^-dependent deacetylase implicated in mitotic checkpoint regulation, metabolic sensing, and tumour suppression; a reduction in its expression would be compatible with, but is not evidence of, the loss of a metabolic checkpoint on anabolic lipid synthesis. *KDM3A* (FDR = 0.059) and *DNMT3B* (FDR = 0.092) reached only the relaxed FDR < 0.10 threshold in the exploratory contrast. Overall, the epigenetic findings are weaker than the lipid-metabolic findings: they did not survive FDR < 0.05 in the confirmatory comparison, and any role for *EHMT2*, *SIRT2*, *KDM3A*, or *DNMT3B* in grade progression remains a hypothesis for testing in larger, adequately powered cohorts.

### 3.5. The Lipid–Epigenetic Connectivity Ranking: A Statistically Robust but Cell-Type-Heterogeneous Co-Expression Pattern

The connectivity ranking placed *FASN*, *KMT2C*, *TET2*, *SETD5*, and *KDM5B* among the most connected genes in the 94-gene lipid–epigenetic matrix. We emphasise that this ranking is correlative and descriptive: it reflects transcriptional co-variation across samples and does not, by itself, indicate physical interaction, shared regulation, or any causal relationship. The absolute connectivity scores are modest in magnitude (mean |r| ≈ 0.26–0.32), so these genes are best regarded as comparatively co-expressed rather than strongly correlated. Importantly, however, a permutation test (10,000 permutations) demonstrated that this co-expression structure is not a chance artefact: all five top-ranked genes had connectivity scores far exceeding the permutation null (null mean 0.111; empirical and family-wise error-corrected *p* < 0.0001), indicating that the ranking is statistically robust even though the individual correlations are modest. With this caveat, the co-ranking of the lipid gene *FASN* with the epigenetic regulators *KMT2C* and *TET2* is of interest as a hypothesis to be tested, because a plausible biological link exists in principle: *FASN*-derived acetyl-CoA can contribute to the cellular acetyl-CoA pool used by histone acetyltransferases, and metabolic flux has been reported to influence chromatin modification states in other systems [26,27]. *KMT2C* is a component of the COMPASS H3K4 methyltransferase complex that deposits H3K4me1/2/3 at active enhancers [28]; its co-expression with lipid metabolic genes is consistent with, but not proof of, a coordinated relationship between enhancer-associated transcription and lipid metabolism, paralleling epigenetic dysregulation patterns reported in osteosarcoma and Ewing sarcoma [24,25,29]. The co-expression of *TET2* is compatible with the established biology of *IDH*-mutant chondrosarcoma, in which D-2HG inhibits α-ketoglutarate-dependent dioxygenases including TET enzymes [30] and has been linked to crosstalk between metabolism and DNA methylation [8,31,32]; however, because *IDH* mutation status was not available for the present cohort (see Section 3.7), this connection remains speculative. An important qualification emerges from the single-cell analysis described below: although the bulk co-expression ranking is statistically robust, the five hub genes are not expressed in the same cell type. *FASN*, *SETD5*, and *KDM5B* are predominantly tumour-cell-derived, whereas *TET2* is expressed mainly in macrophage/myeloid cells and *KMT2C* is not enriched in tumour cells. The bulk lipid–epigenetic connectivity may therefore arise partly from variation in cell-type composition between tumours rather than from a single-cell-autonomous lipid–epigenetic programme. Accordingly, the *FASN*–*KMT2C*–*TET2* association should be interpreted as a statistically supported but cell-type-heterogeneous transcriptional pattern that motivates targeted experimental work, not as an established single-cell mechanistic axis.

### 3.6. Multi-Sample Single-Cell Analysis Localises FASN, SETD5, and KDM5B—But Not KMT2C or TET2—To Tumour Cells

The single-cell RNA-seq analysis of all eight chondrosarcoma samples in GSE184118 (36,012 quality-controlled cells) allows the cell-type distribution of the bulk-derived hub genes to be assessed across multiple tumours rather than a single specimen. Three of the five hub genes—the lipid gene *FASN* and the epigenetic regulators *SETD5* and *KDM5B*—were expressed at significantly higher levels in malignant cells (malignant plus proliferating chondrocytes) than in non-malignant cells (all *p* < 10^−5^; Section 2.8), supporting tumour-cell-predominant expression for these three genes. The remaining two hub genes did not show this pattern: *KMT2C* was not enriched in malignant cells, and *TET2* was expressed most highly in macrophage/myeloid cells rather than in tumour cells, consistent with the well-documented role of *TET2* in the myeloid lineage. This is a more nuanced picture than a single-sample analysis could provide, and it has a direct bearing on the interpretation of the connectivity ranking: although the permutation test shows that the bulk co-expression structure of all five hub genes is statistically robust (Section 2.7), the single-cell data indicate that this co-expression is not explained by uniform co-expression of the five genes within a single cell type. In particular, the bulk-level co-ranking of *FASN* with *TET2* cannot reflect shared tumour-cell-intrinsic regulation, because the two genes are expressed predominantly in different compartments; part of the bulk correlation is therefore likely to arise from variation in tumour-cell content between samples rather than from a tumour-cell-autonomous lipid–epigenetic programme. Within the malignant compartment, hub gene expression stratified by the GEO grade label did not show a consistent monotonic increase with grade, and droplet-based scRNA-seq is in any case subject to substantial gene dropout (the hub genes were detected in only 5–44% of cells); the single-cell data therefore localise *FASN*, *SETD5*, and *KDM5B* expression to tumour cells but do not by themselves establish a grade-dependent gradient at single-cell resolution. Taken together, the multi-sample single-cell analysis refines rather than simply confirms the bulk findings: it supports tumour-cell-predominant expression of the lipid hub gene *FASN* and of *SETD5* and *KDM5B*, while indicating that *KMT2C* and *TET2* are not predominantly tumour-cell-derived and that the *FASN*–epigenetic co-expression detected in bulk data should not be interpreted as a single-cell-resolved mechanistic axis.

### 3.7. Limitations and Future Directions

This study has several important limitations that constrain the strength and generalisability of its conclusions. First, and most fundamentally, the discovery cohort is severely unbalanced and, for two of the four groups, underpowered: the ACT and DDCS groups each comprise only three samples. Any statistical comparison involving these groups is statistically unreliable and biologically inconclusive, and the corresponding effect-size estimates (including the large fold changes reported for *SQLE* and *LDLRAP1* in Grade 3 vs. ACT) are unstable. We therefore restrict the confirmatory conclusions of this study to the Grade 3 vs. Grade 2 comparison, where the sample sizes (*n* = 18 vs. *n* = 29) are more adequate, and treat all ACT- and DDCS-related results as exploratory and hypothesis-generating regardless of their nominal FDR values. The pre-specified relaxed FDR < 0.10 threshold was used only to flag candidate genes for exploratory consideration and does not confer confirmatory status. Second, although the single-cell analysis includes all eight chondrosarcoma samples in GSE184118 rather than a single specimen, it remains limited: eight tumours is still a modest number for assessing inter-patient heterogeneity, the samples are unevenly distributed across grade categories, and droplet-based scRNA-seq is subject to substantial gene dropout, so the single-cell grade comparisons in particular should be interpreted with caution. The single-cell data are best regarded as a cell-type-resolution complement to the bulk analysis rather than an independent confirmatory cohort. Third, the lipid and epigenetic gene panels were assembled by literature-guided curation and were not systematically derived or formally pre-registered, which introduces a risk of selection bias; the panel-based analyses should accordingly be read as a focused, hypothesis-driven survey rather than an unbiased genome-wide screen, and the full gene lists are provided as Supplementary Table S1 for transparency. Fourth, the lipid–epigenetic connectivity analysis is correlative: it identifies transcriptional co-variation, not physical or regulatory interaction, and the absolute correlation values are modest; it is therefore presented as a hypothesis-generating ranking and not as evidence of a mechanistic network. Fifth, although *IDH1*/*IDH2* mutations are central to chondrosarcoma biology, *IDH* mutation status was not annotated in the available GSE299759 metadata. *IDH* mutations are missense point mutations that are not reliably inferable from bulk gene-expression levels, so we did not attempt to call *IDH* status from the RNA-seq data; the inability to stratify by this variable is a genuine limitation, and *IDH*-stratified re-analysis is warranted once appropriately annotated datasets become available. Finally, as a bioinformatic re-analysis of publicly deposited data, this study is correlative by design and cannot establish causal or mechanistic relationships; all proposed mechanisms are hypotheses that require dedicated experimental testing.

Future priorities include the following: (i) experimental validation of *SQLE*, *ACACA*, and *HMGCR* as grade markers via immunohistochemistry in independent adequately powered cohorts; (ii) functional characterisation of the *FASN*–*KMT2C*–*TET2* axis using pharmacological inhibition in chondrosarcoma cell lines combined with ChIP-seq; (iii) evaluation of *EHMT2* inhibitors (e.g., BIX01294, UNC0638) in Grade 3 chondrosarcoma models; (iv) multi-sample scRNA-seq analysis of GSE184118 to determine cell-type specificity across grades; and (v) *IDH* mutation-stratified re-analysis once annotated datasets become available.

## 4. Materials and Methods

### 4.1. Study Design and Data Sources

This study employed a two-cohort design integrating bulk and single-cell RNA sequencing data to characterise lipid metabolic and epigenetic gene expression across the central chondrosarcoma (CCS) grade spectrum and to validate the cell-type specificity of key findings at single-cell resolution. Both datasets are publicly available through the NCBI Gene Expression Omnibus (GEO). No patient-level identifiable data were accessed; ethics approval was not required for this secondary analysis of publicly deposited data.

### 4.2. Bulk RNA-seq Discovery Cohort (GSE299759)

Raw RNA-seq count matrices were retrieved from GEO accession GSE299759, generated by (Leiden University Medical Center, LUMC) from 54 conventional central chondrosarcoma and atypical cartilaginous tumour specimens [7]. Grade and diagnosis metadata were extracted from the accompanying series matrix file (GSE299759_series_matrix.txt). One sample (L4513; phalanx, grade not assignable) was excluded from the grade-stratified analyses, yielding a final cohort of 53 samples: atypical cartilaginous tumour (ACT; *n* = 3), Grade 2 (*n* = 29), Grade 3 (*n* = 18), and dedifferentiated chondrosarcoma (DDCS; *n* = 3). All samples were sequenced on the Illumina NovaSeq 6000 platform (Illumina, Inc., San Diego, CA, USA), and the reads were aligned to the GRCh38/hg38 reference genome using the BioWDL pipeline (LUMC). Raw counts were converted to counts per million (CPM), normalised to library size, and log_2_-transformed (pseudocount = 1). Genes with CPM > 1 in ≥10% of samples were retained, yielding 19,616 expressed genes. The library sizes ranged from 35.4 to 151.5 million reads (cohort median 83.8 M).

### 4.3. Single-Cell RNA-seq Cohort (GSE184118, Eight Chondrosarcomas)

To characterise the cell-type distribution of the bulk-derived hub genes within the chondrosarcoma tumour microenvironment, we analysed single-cell RNA-seq (scRNA-seq) data from GEO accession GSE184118, generated by the Hong Kong group (Commun Biol, 2024) [17]. This dataset comprises 10× Genomics Chromium v3 scRNA-seq profiles from eight conventional central chondrosarcomas of varying grade, one enchondroma, one chondroblastic osteosarcoma, and one foetal femur control. All eight chondrosarcoma samples (L07, L28, L31, L44, L63, L80, L81, L83; GEO grade labels spanning low-, intermediate-, high-grade, and dedifferentiated tumours) were included; the enchondroma, osteosarcoma, and foetal-femur samples were not chondrosarcomas and were excluded. Raw Cell Ranger output files (barcodes.tsv.gz, features.tsv.gz, matrix.mtx.gz) were loaded for each sample. Uniform quality-control filters retained cells with 200–6000 detected genes and a mitochondrial read fraction <25%, yielding 36,012 quality-controlled cells from 37,864 input barcodes across the eight samples. Counts were library-size normalised to 10,000 reads per cell and log-transformed; the 2000 most highly variable genes (selected in a batch-aware manner across samples) were used for principal component analysis (30 components). To correct for inter-sample technical variation, the principal components were integrated across samples using Harmony, and the resulting batch-corrected embedding was used for nearest-neighbour graph construction, Leiden community detection, and UMAP visualisation. Clusters were assigned to cell types using canonical marker genes (malignant chondrocyte: COL2A1, ACAN, SOX9, COL9A1; fibroblast/cancer-associated fibroblast: COL1A1, COL1A2, DCN, LUM; pericyte/mural: RGS5, PDGFRB, NOTCH3; endothelial: PECAM1, VWF, EGFL7; macrophage/myeloid: CD68, CD163, LYZ, AIF1; T/NK cell: CD3D, CD3E, IL7R; proliferating cells: MKI67, TOP2A). Hub gene expression was compared between malignant and non-malignant compartments using Mann–Whitney U tests on log-normalised values.

### 4.4. Curated Gene Panels

A lipid metabolism panel (44 genes) encompassed fatty acid synthesis (*FASN*, *ACACA*, *SCD*, *SCD5*, *ELOVL2*/6), β-oxidation (*ACADM*, *ACADVL*, *ACAD9*, *CPT1A*, *CPT2*, *ECHS1*, *HADHB*), cholesterol metabolism (*HMGCR*, *LDLR*, *PCSK9*, *SQLE*, *DHCR24*, *CETP*, *STARD3*), triglyceride handling (*DGAT2*, *LPL*, *LIPC*, *MTTP*, *ACSL1*, *ACAT1*), lipid droplet proteins (*PLIN1*–4), apolipoproteins (*APOE*, *APOA2*, *APOA5*, *APOC1*), fatty acid-binding proteins (*FABP4*, *FABP5*), and nuclear receptors/transcription factors (*PPARA*, *PPARG*, *PPARD*, *NR1H2*, *NR1H4*, *ATF4*). An epigenetic regulator panel (50 genes) encompassed DNA methyltransferases (*DNMT1*, *DNMT3A*/B/L), TET family demethylases (*TET1*–3), histone methyltransferases (*EZH2*, *KMT2A*/C, *SETD1A*/2/5, *SETDB1*, *EHMT1*/2), histone demethylases (*KDM1A*, *KDM3A*, *KDM4A*–C, *KDM5A*/B, *KDM6B*), acetyltransferases (*KAT2A*/B, *KAT5*, *KAT6A*, *EP300*), histone deacetylases (*HDAC1*–7, *HDAC9*), sirtuins (*SIRT1*–7), and chromatin architectural factors (*CTCF*, *ATRX*, *MYC*, *CBX7*/8, *CEBPB*). Both panels were assembled by literature-guided curation rather than systematic or automated derivation. Lipid metabolism genes were selected to provide representative coverage of the principal functional branches of cellular lipid metabolism as defined in established reviews of lipid metabolic reprogramming in cancer [9,10,11,12,13,14], and epigenetic regulators were selected to span the major classes of DNA- and histone-modifying enzymes implicated in mesenchymal and bone tumours [24,25,29,33]. The panels were defined a priori, before any differential expression or hub analysis, but were not formally pre-registered. We acknowledge that researcher-curated panels of this type carry a risk of selection bias, and that genes outside these panels may also contribute to the processes studied; the panel-based results should therefore be interpreted as a focused, hypothesis-driven survey rather than an unbiased genome-wide screen. The full gene lists are provided in Supplementary Table S1 to allow independent scrutiny and re-analysis.

### 4.5. Bulk RNA-seq Analytical Methods

Principal component analysis (PCA) was performed on all expressed genes after per-gene standardisation (scikit-learn v1.3). Grade-restricted PCAs were performed separately on the 44-gene lipid and 50-gene epigenetic panels. Heatmaps were generated according to the z-scoring expression per gene (clipped to [−3, +3]), with the samples ordered according to histological grade and genes according to Ward-linkage hierarchical clustering (seaborn v0.12). Composite lipid and epigenetic expression scores were computed as the mean of the per-gene z-scores across all panel members per sample; grade-group differences in composite scores were tested using Kruskal–Wallis H-tests. For gene-level differential expression, a two-stage statistical approach was employed: a Kruskal–Wallis H-test applied across all four grade groups as an omnibus test of heterogeneity (BH correction), followed by pairwise post hoc comparisons using the Dunn test (z-score method, BH correction within each pairwise contrast). Because the ACT and DDCS groups each contained only three samples, the Grade 3 vs. Grade 2 contrast was pre-specified as the primary, adequately powered comparison, and confirmatory conclusions were restricted to this contrast; all pairwise comparisons involving the ACT or DDCS groups were designated a priori as exploratory and underpowered, and are reported as such regardless of nominal FDR value. The primary significance threshold was FDR < 0.05 and |log_2_FC| > 0.5. A pre-specified relaxed secondary threshold of FDR < 0.10 was applied only to flag biologically plausible findings for exploratory consideration. For the lipid–epigenetic interface, an exploratory connectivity ranking was computed: each gene was assigned a connectivity score equal to its mean absolute Pearson correlation coefficient (|r|) with all other 93 genes in the 94-gene combined lipid–epigenetic matrix, encompassing both within-module (lipid–lipid, epigenetic–epigenetic) and between-module (lipid–epigenetic) correlations, and genes were ranked by this score. This ranking is a descriptive, hypothesis-generating device used only to prioritise genes for discussion; no fixed |r| cut-off was used to define statistically significant interactions, and the absolute |r| values are modest and should not be read as evidence of strong or direct co-regulation. To assess whether the connectivity scores of the top-ranked genes exceeded what would be expected by chance, a permutation test was performed: the expression values of each gene were independently permuted across the 53 samples, breaking inter-gene correlation while preserving each gene’s marginal distribution, and connectivity scores were recomputed; this procedure was repeated 10,000 times to construct a null distribution. Per-gene empirical *p*-values were defined as the proportion of permutations in which the permuted connectivity score equalled or exceeded the observed value, and a family-wise error-controlled *p*-value was additionally computed by comparing each observed score with the distribution of per-permutation maximum scores. All bulk RNA-seq analyses were implemented in Python 3.11 (numpy 1.24, pandas 2.0, scipy 1.11, matplotlib 3.7, seaborn 0.12, scikit-learn 1.3).

### 4.6. Data and Code Availability

All primary data are publicly available: bulk RNA-seq data under GEO accession GSE299759 (https://www.ncbi.nlm.nih.gov/geo/query/acc.cgi?acc=GSE299759, accessed on 1 March 2026); single-cell RNA-seq data under GEO accession GSE184118 (https://www.ncbi.nlm.nih.gov/geo/query/acc.cgi?acc=GSE184118, accessed on 1 March 2026). The analysis code is available from the corresponding author upon reasonable request.

## 5. Conclusions

This study analysed bulk RNA-seq data from 53 graded central chondrosarcoma samples (GSE299759), complemented by a single-cell RNA-seq analysis of eight chondrosarcomas (GSE184118; 36,012 cells). In the primary, adequately powered Grade 3 vs. Grade 2 comparison, Kruskal–Wallis omnibus testing followed by Dunn post hoc analysis identified upregulation of de novo lipogenesis genes (*SQLE*, *ACACA*, *FASN*) at FDR < 0.05; this constitutes the confirmatory finding of the study. Additional lipid and epigenetic differences (including *HMGCR*, *LDLRAP1*, *EHMT2*, and *SIRT2*) were observed only in comparisons involving the ACT or DDCS groups, each limited to three samples, and are therefore reported as exploratory. A lipid–epigenetic connectivity ranking highlighted *FASN*, *KMT2C*, and *TET2*; a permutation test confirmed that this co-expression structure is statistically robust (*p* < 0.0001), while the multi-sample single-cell analysis showed that the hub genes are not uniformly tumour-cell-derived—*FASN*, *SETD5*, and *KDM5B* are predominantly expressed in malignant cells, whereas *KMT2C* and *TET2* are not. Overall, these transcriptomic associations nominate *SQLE*, *ACACA*, and *FASN* as candidate genes for experimental investigation in adequately powered, *IDH*-annotated cohorts, and indicate that the lipid–epigenetic connectivity should be interpreted as a statistically supported but cell-type-heterogeneous transcriptional pattern rather than a single-cell-autonomous mechanistic axis.

## Figures and Tables

**Table 1:** Study cohort characteristics by histological grade.

Characteristic	ACT (*n* = 3)	Grade 2 (*n* = 29)	Grade 3 (*n* = 18)	DDCS (*n* = 3)
Histological class	ACT	Conventional CS	Conventional CS	Dedifferentiated CS
Library size range (M reads)	61.2–107.8	35.4–151.5	41.3–148.6	55.9–122.4
Median library size (M reads)	84.5	83.6	83.9	85.1
Expressed genes (CPM > 1, after QC)	17,842	19,616	18,904	18,211
Cohort-wide technical parameters (all groups)				
Sequencing platform: Illumina NovaSeq 6000				
Genome assembly: GRCh38/hg38				
Read alignment/quantification: STAR/featureCounts (GSE299759)				
Normalisation method: CPM + log_2_(CPM + 1)				
GEO accession: GSE299759 | PMID: 40976495|Meijer et al., 2025 [7]				

ACT, atypical cartilaginous tumour; DDCS, dedifferentiated chondrosarcoma; CS, chondrosarcoma.

**Table 2:** Differential expression summary for lipid and epigenetic panels across grade comparisons.

Comparison	Panel	Tested	FDR < 0.05	FDR < 0.10	Sig. Genes FDR < 0.05	Additional FDR < 0.10 *
Grade 3 vs. ACT	Lipid	44	4	8	*SQLE* (↑), *ACACA* (↑), *HMGCR* (↑), *LDLRAP1* (↑)	*FASN* (↑), *SCD5* (↑), *CETP* (↓), *PPARG* (↓)
Grade 3 vs. ACT	Epigenetic	49	2	4	*EHMT2* (↑), *SIRT2* (↓)	*KDM3A* (↑), *DNMT3B* (↑)
Grade 3 vs. Grade 2	Lipid	44	3	4	*SQLE* (↑), *ACACA* (↑), *FASN* (↑)	*SCD5* (↑)
Grade 3 vs. Grade 2	Epigenetic	49	0	2	—	*EHMT2* (↑), *DNMT3B* (↑)
DDCS vs. ACT †	Lipid	44	4	7	*ACACA* (↑), *FASN* (↑), *MTTP* (↓), *ELOVL6* (↑)	*APOE* (↓), *ATF4* (↓), *SQLE* (↑)
DDCS vs. ACT †	Epigenetic	49	2	7	*DNMT3B* (↑), *SIRT2* (↓)	*KDM5B* (↓), *SETD5* (↑), *KMT2C* (↑), *ATRX* (↓), *EHMT2* (↑)

* Significant at FDR < 0.05 (BH correction, Dunn post hoc). FDR < 0.10 = pre-specified exploratory threshold. † DDCS vs. ACT comparison: both groups *n* = 3; all results exploratory regardless of FDR tier. Test: Kruskal–Wallis omnibus (four groups) followed by Dunn post hoc pairwise (BH correction). ↑, upregulated; ↓, downregulated.

**Table 3:** Top 15 hub genes ranked by mean |Pearson r| across the 94-gene lipid–epigenetic co-expression matrix.

Rank	Gene	Category	Biological Function
1	*KMT2C*	Epigenetic—H3K4 methyltransferase	H3K4me1/2/3 writer; active enhancer marking
2	*KDM5B*	Epigenetic—H3K4 demethylase	H3K4me3 eraser; transcriptional repression
3	*SETD5*	Epigenetic—H3K36 methyltransferase	Transcription elongation; H3K36me
4	*TET2*	Epigenetic—DNA demethylase	5mC → 5hmC conversion; *IDH*-sensitive
5	*FASN*	Lipid—fatty acid synthesis	De novo lipogenesis; acetyl-CoA to palmitate
6	*HDAC4*	Epigenetic—class IIa HDAC	Histone deacetylation; MEF2 co-repressor
7	*SETDB1*	Epigenetic—H3K9 methyltransferase	H3K9me3 silencing; heterochromatin
8	*KDM6B*	Epigenetic—H3K27 demethylase	H3K27me3 eraser; Polycomb antagonism
9	*KAT5*	Epigenetic—acetyltransferase	H4K16ac; DNA damage response
10	*TET1*	Epigenetic—DNA demethylase	5mC oxidation; gene body demethylation
11	*PLIN3*	Lipid—lipid droplet scaffold	Lipid droplet biogenesis; TAG mobilisation
12	*PPARA*	Lipid—nuclear receptor	FA oxidation; peroxisomal gene activation
13	*ATF4*	Lipid/ISR—transcription factor	ISR; lipid–epigenetic interface; *FASN* co-regulator
14	*ACACA*	Lipid—fatty acid synthesis	Malonyl-CoA synthesis; lipogenesis gate
15	*SQLE*	Lipid—cholesterol synthesis	Squalene epoxidase; rate-limiting sterol step

Hub score = mean |Pearson r| with all other 93 genes in the combined matrix.

## Data Availability

All primary data are publicly available. Bulk RNA-seq data: GEO accession GSE299759 (https://www.ncbi.nlm.nih.gov/geo/query/acc.cgi?acc=GSE299759, accessed on 1 March 2026). Single-cell RNA-seq data: GEO accession GSE184118 (https://www.ncbi.nlm.nih.gov/geo/query/acc.cgi?acc=GSE184118, accessed on 1 March 2026). Analysis code available from the corresponding author upon reasonable request.

## Supplementary Materials

The following supporting information can be downloaded at: https://www.mdpi.com/article/10.3390/ijms27125307/s1.
