# Supplementary material for: Lipid Metabolic Reprogramming and Epigenetic Co-Dysregulation Across the Central Chondrosarcoma Grade Spectrum: A Multi-Cohort RNA-seq Study
Source: Int J Mol Sci. 2026 Jun 11;27(12):5307. doi: 10.3390/ijms27125307 (PMC13299284; doi:10.3390/ijms27125307)
Supplement: Supplementary file 1 [file ijms-27-05307-s001.zip › ijms-4256794-supplementary.pdf]

Supplementary Material

Lipid Metabolic Reprogramming and Epigenetic Co-dysregulation Across the Central Chondrosarcoma Grade Spectrum: A Multi-Cohort RNA-seq Study

International Journal of Molecular Sciences | Manuscript ID: ijms-4256794

Supplementary Table S1. Lipid metabolism gene panel

n = 44 genes. Panel assembled by literature-guided curation (see Materials and Methods, Section 2.4).

No.	Gene symbol	Functional category	Brief functional role
1	**FASN**	Fatty acid synthesis	Fatty acid synthase; de novo palmitate synthesis
2	**ACACA**	Fatty acid synthesis	Acetyl-CoA carboxylase alpha; malonyl-CoA synthesis
3	**SCD**	Fatty acid synthesis	Stearoyl-CoA desaturase; monounsaturated fatty acid synthesis
4	**SCD5**	Fatty acid synthesis	Stearoyl-CoA desaturase 5; desaturation of fatty acids
5	**ELOVL2**	Fatty acid synthesis	Fatty acid elongase 2; long-chain PUFA elongation
6	**ELOVL6**	Fatty acid synthesis	Fatty acid elongase 6; C16-C18 fatty acid elongation
7	**ACADM**	Fatty acid beta-oxidation	Medium-chain acyl-CoA dehydrogenase
8	**ACADVL**	Fatty acid beta-oxidation	Very-long-chain acyl-CoA dehydrogenase
9	**ACAD9**	Fatty acid beta-oxidation	Acyl-CoA dehydrogenase family member 9
10	**CPT1A**	Fatty acid beta-oxidation	Carnitine palmitoyltransferase 1A; mitochondrial FA import
11	**CPT2**	Fatty acid beta-oxidation	Carnitine palmitoyltransferase 2; mitochondrial FA import
12	**ECHS1**	Fatty acid beta-oxidation	Enoyl-CoA hydratase; mitochondrial beta-oxidation
13	**HADHB**	Fatty acid beta-oxidation	Trifunctional enzyme beta subunit; beta-oxidation
14	**HMGCR**	Cholesterol metabolism	HMG-CoA reductase; rate-limiting mevalonate pathway enzyme
15	**LDLR**	Cholesterol metabolism	Low-density lipoprotein receptor; cholesterol uptake
16	**PCSK9**	Cholesterol metabolism	Proprotein convertase; LDLR degradation
17	**SQLE**	Cholesterol metabolism	Squalene epoxidase; rate-limiting sterol biosynthesis step
18	**DHCR24**	Cholesterol metabolism	24-dehydrocholesterol reductase; cholesterol synthesis
19	**CETP**	Cholesterol metabolism	Cholesteryl ester transfer protein; lipid exchange
20	**STARD3**	Cholesterol metabolism	StAR-related lipid transfer protein 3; cholesterol transport
21	**DGAT2**	Triglyceride handling	Diacylglycerol O-acyltransferase 2; triglyceride synthesis
22	**LPL**	Triglyceride handling	Lipoprotein lipase; triglyceride hydrolysis
23	**LIPC**	Triglyceride handling	Hepatic lipase C; lipoprotein metabolism
24	**MTTP**	Triglyceride handling	Microsomal triglyceride transfer protein; lipoprotein assembly
25	**ACSL1**	Triglyceride handling	Acyl-CoA synthetase long-chain 1; fatty acid activation
26	**ACAT1**	Triglyceride handling	Acetyl-CoA acetyltransferase 1; ketone/lipid metabolism
27	**PLIN1**	Lipid droplet protein	Perilipin 1; lipid droplet scaffold
28	**PLIN2**	Lipid droplet protein	Perilipin 2; lipid droplet biogenesis
29	**PLIN3**	Lipid droplet protein	Perilipin 3; lipid droplet biogenesis and TAG mobilisation
30	**PLIN4**	Lipid droplet protein	Perilipin 4; lipid droplet coat protein
31	**APOE**	Apolipoprotein	Apolipoprotein E; lipoprotein transport
32	**APOA2**	Apolipoprotein	Apolipoprotein A-II; HDL component
33	**APOA5**	Apolipoprotein	Apolipoprotein A-V; triglyceride metabolism regulator
34	**APOC1**	Apolipoprotein	Apolipoprotein C-I; lipoprotein metabolism
35	**FABP4**	Fatty acid-binding protein	Fatty acid-binding protein 4; intracellular FA transport
36	**FABP5**	Fatty acid-binding protein	Fatty acid-binding protein 5; intracellular FA transport
37	**PPARA**	Nuclear receptor / transcription factor	PPAR alpha; fatty acid oxidation gene activation
38	**PPARG**	Nuclear receptor / transcription factor	PPAR gamma; lipid catabolism and adipogenesis regulator
39	**PPARD**	Nuclear receptor / transcription factor	PPAR delta; fatty acid metabolism regulator
40	**NR1H2**	Nuclear receptor / transcription factor	Liver X receptor beta; sterol-sensing nuclear receptor
41	**NR1H4**	Nuclear receptor / transcription factor	Farnesoid X receptor; bile acid / lipid signalling
42	**ATF4**	Nuclear receptor / transcription factor	Activating transcription factor 4; ISR and lipogenic co-regulator
43	**LDLRAP1**	Cholesterol metabolism	LDL receptor adaptor protein 1; clathrin-dependent LDLR internalisation
44	**DGAT1**	Triglyceride handling	Diacylglycerol O-acyltransferase 1; triglyceride synthesis

Supplementary Table S1. Epigenetic regulator gene panel

n = 50 genes. Panel assembled by literature-guided curation (see Materials and Methods, Section 2.4).

No.	Gene symbol	Functional category	Brief functional role
1	**DNMT1**	DNA methyltransferase	Maintenance DNA methyltransferase
2	**DNMT3A**	DNA methyltransferase	De novo DNA methyltransferase 3A
3	**DNMT3B**	DNA methyltransferase	De novo DNA methyltransferase 3B
4	**DNMT3L**	DNA methyltransferase	DNMT3-like; regulatory cofactor for de novo methylation
5	**TET1**	DNA demethylase	Ten-eleven translocation 1; 5mC to 5hmC oxidation
6	**TET2**	DNA demethylase	Ten-eleven translocation 2; 5mC oxidation, IDH-sensitive
7	**TET3**	DNA demethylase	Ten-eleven translocation 3; 5mC oxidation
8	**EZH2**	Histone methyltransferase	Enhancer of zeste 2; H3K27me3 writer, PRC2 catalytic subunit
9	**KMT2A**	Histone methyltransferase	Lysine methyltransferase 2A (MLL1); H3K4 methylation
10	**KMT2C**	Histone methyltransferase	Lysine methyltransferase 2C; H3K4me1/2/3, enhancer marking
11	**SETD1A**	Histone methyltransferase	SET domain containing 1A; H3K4 methylation
12	**SETD2**	Histone methyltransferase	SET domain containing 2; H3K36me3 writer
13	**SETD5**	Histone methyltransferase	SET domain containing 5; transcription elongation, H3K36me
14	**SETDB1**	Histone methyltransferase	SET domain bifurcated 1; H3K9me3 silencing, heterochromatin
15	**EHMT1**	Histone methyltransferase	Euchromatic histone methyltransferase 1 (GLP); H3K9me1/2
16	**EHMT2**	Histone methyltransferase	Euchromatic histone methyltransferase 2 (G9a); H3K9me1/2
17	**KDM1A**	Histone demethylase	Lysine demethylase 1A (LSD1); H3K4/H3K9 demethylation
18	**KDM3A**	Histone demethylase	Lysine demethylase 3A; H3K9me1/2 demethylation
19	**KDM4A**	Histone demethylase	Lysine demethylase 4A; H3K9me3/H3K36me3 demethylation
20	**KDM4B**	Histone demethylase	Lysine demethylase 4B; H3K9me3 demethylation
21	**KDM4C**	Histone demethylase	Lysine demethylase 4C; H3K9me3 demethylation
22	**KDM5A**	Histone demethylase	Lysine demethylase 5A; H3K4me3 demethylation
23	**KDM5B**	Histone demethylase	Lysine demethylase 5B; H3K4me3 eraser, transcriptional repression
24	**KDM6B**	Histone demethylase	Lysine demethylase 6B; H3K27me3 eraser, Polycomb antagonism
25	**KAT2A**	Histone acetyltransferase	Lysine acetyltransferase 2A (GCN5); histone acetylation
26	**KAT2B**	Histone acetyltransferase	Lysine acetyltransferase 2B (PCAF); histone acetylation
27	**KAT5**	Histone acetyltransferase	Lysine acetyltransferase 5 (TIP60); H4K16ac, DNA damage response
28	**KAT6A**	Histone acetyltransferase	Lysine acetyltransferase 6A (MOZ); H3 acetylation
29	**EP300**	Histone acetyltransferase	E1A-binding protein p300; H3K27ac writer, transcriptional coactivator
30	**HDAC1**	Histone deacetylase	Histone deacetylase 1; class I HDAC
31	**HDAC2**	Histone deacetylase	Histone deacetylase 2; class I HDAC
32	**HDAC3**	Histone deacetylase	Histone deacetylase 3; class I HDAC
33	**HDAC4**	Histone deacetylase	Histone deacetylase 4; class IIa HDAC, MEF2 co-repressor
34	**HDAC5**	Histone deacetylase	Histone deacetylase 5; class IIa HDAC
35	**HDAC6**	Histone deacetylase	Histone deacetylase 6; class IIb HDAC, cytoplasmic
36	**HDAC7**	Histone deacetylase	Histone deacetylase 7; class IIa HDAC
37	**HDAC9**	Histone deacetylase	Histone deacetylase 9; class IIa HDAC
38	**SIRT1**	Sirtuin	Sirtuin 1; NAD+-dependent deacetylase, metabolic sensing
39	**SIRT2**	Sirtuin	Sirtuin 2; NAD+-dependent deacetylase, cytoplasmic
40	**SIRT3**	Sirtuin	Sirtuin 3; mitochondrial NAD+-dependent deacetylase
41	**SIRT4**	Sirtuin	Sirtuin 4; mitochondrial ADP-ribosyltransferase
42	**SIRT5**	Sirtuin	Sirtuin 5; mitochondrial desuccinylase/demalonylase
43	**SIRT6**	Sirtuin	Sirtuin 6; chromatin-associated NAD+-dependent deacetylase
44	**SIRT7**	Sirtuin	Sirtuin 7; nucleolar NAD+-dependent deacetylase
45	**CTCF**	Chromatin architectural factor	CCCTC-binding factor; chromatin insulator and looping
46	**ATRX**	Chromatin architectural factor	ATRX chromatin remodeller; heterochromatin maintenance
47	**MYC**	Chromatin architectural factor	MYC proto-oncogene; transcriptional amplifier
48	**CBX7**	Chromatin architectural factor	Chromobox 7; Polycomb (PRC1) component, H3K27me3 reader
49	**CBX8**	Chromatin architectural factor	Chromobox 8; Polycomb (PRC1) component
50	**CEBPB**	Chromatin architectural factor	CCAAT/enhancer-binding protein beta; transcription factor
